# Insane in Foreign Countries

**Published:** 1889-07

**Authors:** 


THE INSANE IN FOREIGN COUNTRIES, by William P. Letchworth, President of the New York State Board of Charities. New York and London, G. P. Putnam's Sons. The Knickerbocker Press, 1889.

The above is a large volume of 365 octavo pages, well gotten out in cloth, and beautifully illustrated.

The work is an outcome of an investigation of foreign charitable institutions, pursued without interruption for seven months, during which time special attention was given to various kinds of provision made for the insane poor. The information is now published in the hope of furthering the elucidation of a subject which materially concerns not only the State as a whole, but each one of its citizens.

As the information which this work contains was in a large measure obtained from learned and experienced specialists, it must not only be regarded as the latest and most advanced, but as entitled to the highest confidence of the reader.

The countries visited are England, Ireland, Continental Countries. The Colony of Gheel and the Provincial Insane Asylum of Alt-Scherbitz.

In concluding part of the volume the author gives a general survey or "*Resume*" of the entire field of observation especially suggestive and practical on the following points, viz.: Location, buildings, furnishing and decoration, Orderly arrangement, Grounds, Sewage, Greater freedom, Nurses trained, Medical officers, Attendants, Night service, Religious exercises, Amusements, employment, Renumeration, Dress and clothing, Dietary and dining rooms, Alcoholic stimulants, Visitations, Letters, Admission and discharge, Summer resorts, Criminal insane, Boarding out, Poor houses, State wards, Public accounts, Supervision, etc.

